# Ethnobotanical Inventory on the Vernacular Names of Useful Plants: Data of over 120 Years for the Ligurian Region (Northern Italy)

**DOI:** 10.3390/plants15142193

**Published:** 2026-07-17

**Authors:** Luigi Minuto, Chiara Marescalchi, Laura Cornara

**Affiliations:** 1 Department of Earth, Environmental and Life Sciences (DISTAV), University of Genova, Corso Europa 26, 16132 Genova, Italy; luigi.minuto@unige.it (L.M.); chia.marescalchi@gmail.com (C.M.); 2 PLANTA/Center for Research, Documentation and Training, Via Serraglio Vecchio 28, 90123 Palermo, Italy

**Keywords:** folk botanical knowledge, vernacular flora, biocultural heritage, cultural practices, ethnobotanical indices

## Abstract

Knowledge of vernacular plant names is fundamental for ethnobotanical research, aiding both in the identification of botanical taxa and the preservation of linguistic and ethnological heritage. This study examines the Ligurian vernacular plant names recorded in botanical publications from the late 19th century to 2020, spanning over 120 years. Beyond contributing to historical Italian ethnobotany, the aim was to describe how communities perceived and utilized plants during this long period, which saw significant socioeconomic shifts and historical events, such as the two World Wars. The main results show that the vernacular flora quotes a total of 9474 citations of dialectal names referring to 950 species, corresponding to 2120 basic vernacular names. Overall, 32.27% of wild taxa were sufficiently well-known to have a vernacular name, highlighting a good conservation of botanical knowledge for the Ligurian flora, characterized by very high biodiversity. However, a comparison between the periods before and after World War II showed a decline in the number of both cited species (30% reduction) and vernacular names recorded (14.48% reduction). These data should be viewed as merely indicative, given that they were collected using methodologies that were not always consistent across the different time periods. Nevertheless, it can be hypothesized that socioeconomic and land use changes—occurring over such a long and complex historical period—have contributed to reducing the population’s reliance on wild plants, thereby diminishing knowledge regarding their vernacular names. Ultimately, our survey explored how Liguria’s folk botanical knowledge has survived to this day, in an effort to provide a foundation for safeguarding this cultural heritage.

## 1. Introduction

The field of research that concerns the inter-relationships between humans and plants in the past is Historical Ethnobotany [1]. In this context, the knowledge of the vernacular names of plants in different geographical and cultural areas plays an important role in the identification of plant species, linking such names to specific botanical taxa. Furthermore, this cultural heritage also has a significant linguistic and ethnological value, highlighting the strong connection between humans and the surrounding nature, including the utilitarian uses of plants by human societies throughout history. These uses are also closely linked to the cultural traditions and beliefs of each ethnic group in different geographical areas of the world; therefore, different names may assign to the same plant in different languages and regions [2]. As regards Italy, already in 1924, the famous botanist Otto Penzig had highlighted that the vernacular names attributed to plants vary considerably, depending on the Italian region considered and its local dialect, and even within different areas of the same region. An example is provided by the strawberry tree, *Arbutus unedo* L., known in Italy with over 90 different names of heterogeneous roots, including: *albatro*, *albatrello*, *urlo*, *rossello* in Tuscany; *armùn*, *armuin*, *arbüssaru*, *murta* in Liguria; *frola marina*, *frole d’Natal* in Piedmont; *fraghe de montagna*, *cornarolo* in Veneto; *lallerone*, *allerone* in Umbria; *cacùmmaro*, *imbriachi* in Calabria; *mbriàculu*, *miriaculi* in Sicily; and *olidone*, *ghilisoni* in Sardinia [3]. It should be remembered, however, that calling the same plant by another name does not alter its properties or modify its traditional uses. Even the popular adage from Shakespeare’s “Romeo and Juliet” (1594–1597) emphasized that “A rose by any other name would smell as sweet” affirming that the names of things—in this case, referring to famous flowers—do not affect what they actually are. In the above-mentioned case of the strawberry tree, despite the different names by which it is called in the various areas of Italy, its main uses, which concern the medicinal and food fields, are practiced indiscriminately throughout Italy [4].

The origin of the vernacular names of plants can be inspired by various factors, for example, the flowering period, a characteristic shape, a typical smell or taste, the similarity of some plant organs to animal ones (e.g., tail, foot, ear, etc.) and frequently also by the medicinal or magic properties attributed to them in folk medicine, according to previous reports for other Italian regions [5] as well as for other European countries [6].

For example, according to Penzig [3], *Artemisia absinthium* L. is known in northern Italy by various vernacular names, referring to its multiple healing properties that have contributed to its fame as a panacea, e.g., *erba mêgu* and *bun meigu* in Liguria; *erba meja* and *medighet* in Piedmont; *medeghetto* in Lombardia; *medego maïstro* in Veneto; *medegh* in Emilia: all names that refer to the plant as a good doctor (doctor = medico, in Italian). Another example is provided by *Pinguicula vulgaris* L. which is often called by names that refer to the well-known healing properties of its greasy leaves, e.g., *erba unta* in Liguria; *erba olearia* or *erbo de la tailleure* in Piedmont; *erba de tài* in Lombardia; *erba del tajo* in Veneto; and *erba oliosa* in Emilia. These vernacular names derive from the word oil/unctuous (olio/untuoso, in Italian) or from the word cut/wound (taglio/ferita, in Italian).

Finally, many vernacular names used in Italy are influenced by neighboring countries (France, Germany, etc.) or have roots that recall long periods of foreign domination: for example, *dafni*, *dàfina* or *dafli* used for *Laurus nobilis* L. in Calabria derives from the Greek term *dáphnē* (name of the nymph associated with the laurel tree); the name *fastuca* used in Sicily for *Pistacia vera* L. derives from the Arabic term *fastûq* (pistachio), while *giunivert* used in Sardinia for *Petroselinum crispum* (Mill.) Fuss comes from the Catalan term *julivert* (parsley) [3,7].

Another problem already raised by Penzig himself [3], who pointed out that in different Italian regions the same name could be used for a set of very different plant species, first those that are collected around the summer solstice, or the feast of St. John the Baptist (on June 24th). This is the case of the plants called with the common name of *erba di San Giovanni* (St. John’s Wort) including even species of different genus and family, such as *Hypericum perforatum* L., *Hylotelephium maximum* (L.) Holub, *Verbena officinalis* L., *Lavandula latifolia* Medik., *Rumex acetosa* L. and *Rhinanthus crista-galli* L.

Similarly, Kalle and Sõukland [8] in their recent study on the folk categorization of nature in preliterate societies in Europe, analyzing the local plant names reported in the old ethnobotanical manuscripts and herbaria from Baltic [9,10], highlighted that “a single plant species was not as important as a plant use group”, e.g., species that could be functionally interchangeable. In the same context, they reported that also in their survey the plants whose flowering was associated with Midsummer’s Day were all called “St. John’s wort.”

It should also be emphasized that the difficulty of ascertaining the identity of a plant species by its vernacular name is common to many European countries, as highlighted by Wróbel [11], referring to the case that “…a general Slavic name denotes different plants in different Slavic languages”. Several examples are provided in the subsequent study conducted by Waniakowa [12], who pointing out that “…in all Slavic dialects as well as in the history of Slavic languages we can find cases where a given name may refer to several species of plant and a particular species may have up to several dozen names”.

Finally, another issue is that more recent ethnobotanical research has been conducted according to international standards and validated methods, while research into historical folk botanical knowledge has often employed uneven methodology and pursued diverse objectives [13]. Furthermore, these historical data have been the subject of limited studies, primarily focusing medicinal plants [14,15] or wild vegetables [16,17], whereas the vernacular names of plants with other uses—handicrafts, agriculture, household or for different utility products—were generally reported less frequently both for Italy [18,19] and other world countries [20,21].

Despite all the complex issues related to vernacular plant names, it is worth noting that they are rapidly disappearing today, along with the culture and traditions of every ethnic group around the world. Therefore, it is crucial that this knowledge be recorded, preserved and documented before it is lost forever [2]. Therefore, we deemed it useful to analyze all documents produced by botanists, in which the vernacular names of Ligurian plants were recorded—albeit using methodologies that were not always directly comparable. The period covered by our survey spans from the late 19th century [22] to 2020 [23], representing a timeframe of 123 years, a period marked by two world wars and major socio-economic changes. This survey aims to document a significant portion of Liguria’s biocultural heritage, which had never before been consolidated into a comprehensive overview spanning more than a century. Other than preserving vernacular plant names and fostering their intergenerational transmission, the data collected can also prove useful for understanding how communities perceived, maintained and passed down their folk botanical knowledge.

## 2. Results

### 2.1. Natural Flora (NF)

The NF quotes a total amount of 2944 taxa (2754 autochthonous and 190 exotics of which 43 cultivated as food and 144 with decorative purposes). The NF has been grouped according to the geographical heterogeneity of the regional territory as follows: 1772 (western, W), 2172 (central, C) and 1514 (eastern, E).

### 2.2. Ethnobotanical Data

The selected ethnobotanical literature of Liguria, belonging to 30 bibliographic sources, quotes a total amount of 9724 plant citations belonging to 950 species and 126 families.

### 2.3. Ethnobotanical Flora (EF)

The EF includes 628 taxa (2 Lichens, 12 Pteridophyta, 9 Gymnospermae, 605 Angiospermae; 434 herbs, 21 bulbous, 35 shrubs, 65 bushes, 73 trees) defined with a specific use.

The Herbarium specimens of Ligurian plants are deposited at the Herbarium Universitatis Genuensis (official international acronym: GE) or at the herbarium of the Giacomo Doria Civic Museum of Natural History (official international acronym: GDOR), both located in Genova, Italy.

According to the taxonomical analysis of EF the families with more than 10 species with ethnobotanical uses are 27 (Table 1) and the five more used families (>40 applications) are: Asteraceae, Fabaceae, Lamiaceae, Poaceae and Rosaceae.

**Figure 1:**
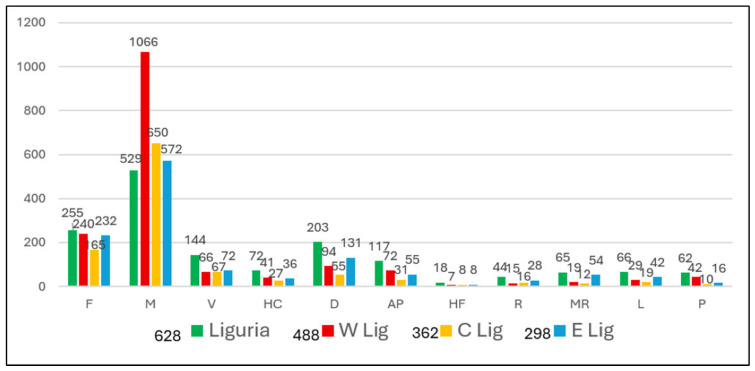
Ethnobotanical flora and subdivision in the eleven categories of different applications. F = food, M = medicinal, V = veterinary, HC = handcraft, D = domestic, AP = agro-pastoral, HF = fishing, R = religious, MR = magical-ritual, L = ludic, P = poison.

However, most plants of EF frequently have multiple usefulness, as shown in S1, Supplementary Materials and in Table A1 in Appendix A, and they show variable applications in the three different parts of the region (Figure 1). Most plants are used as medicinal (529 uses), more frequently than as food (255). A third important application is for domestic purpose (203), followed by the agropastoral one (117).

As reported in Table 1, the number of plant families used is always around a hundred (Liguria = 122, W = 112, C = 102, E = 96) but everywhere the families with the greatest number of uses are 27. Among them, the five more used families are: Asteraceae (119), Fabaceae (60), Lamiaceae (56), Rosaceae (53) and Poaceae (43). About 11.94% of plants belonging to EF have a RU ≥ 5 (29 taxa with RU = 5, 20 = 6, 11 = 7, 10 = 8). The higher value of use report (RU = 9) is found for *Arundo donax* L., *Castanea sativa* Mill., *Juniperus communis* L., *Olea europaea* L., *Salvia rosmarinus* Schleid and *Sambucus nigra* L.

EI having a regional value of 20.03 shows a more evident variability of the traditional use of local flora among the different parts of the territory: W = 27.54, C = 16.67, E = 19.68 (Table 2).

EImed (Table 2) shows a strongly different use of medicinal plants in the region, with the higher value in the western zone (22.80), much higher than in the other two parts of the territory (C = 12.48; E = 13.41) and in the whole region (16.87).

### 2.4. Vernacular Flora (VF) and Phytonomys

**Figure 2:**
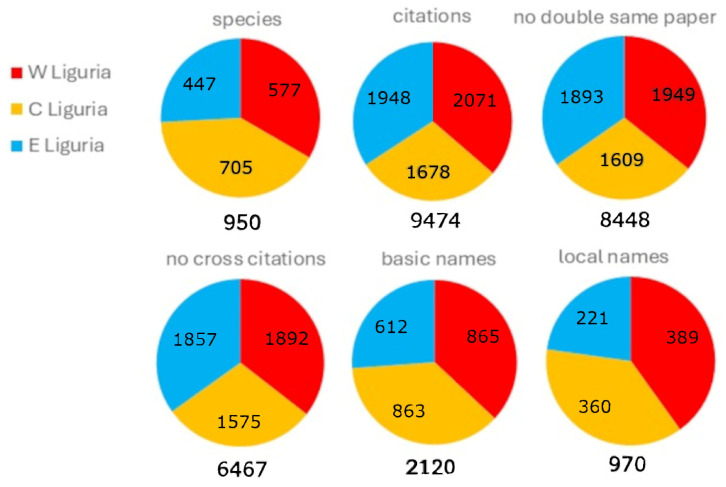
Geographical distribution of vernacular flora. In bold the value for the whole region.

The VF quotes a total amount of 9724 vernacular (=dialectal) names citations belonging to 950 species (Figure 2) of which 913 are spontaneous and 133 are usually cultivated (and among these 93 are horticultural crops from wild species). Because of the selection made by eliminating repeated names used in different villages of the same study area, VF decreased to 8448; purging multiple cross-referred names quoted in different studies, they have further reduced to 6467. The detection of basic names without any local alteration and variation yields a total of only 2120 items.

The analysis of the geographical distribution of names (Figure 2) indicates a greater number of locally used basic names in the western and central parts of the region than in the eastern part (865 and 863 vs. 612, respectively). Similarly, the number of names used exclusively in one part of the region decreases from west to east (389, 360, 221).

EPI, showing the traditional knowledge of local species (Table 2), is always about 0.30 if related to both the whole region and the subdivisions of the territory (W = 0.33; C = 0.32; E = 0.29).

The VF identified 25 taxa associated with more than 30 names (Table 3). These are very common species, mostly wild greens or cultivated plants. The highest ratio of vernacular names to uses (3.81%) was observed for *Reichardia picroides* (L.) Roth.

**Figure 3:**
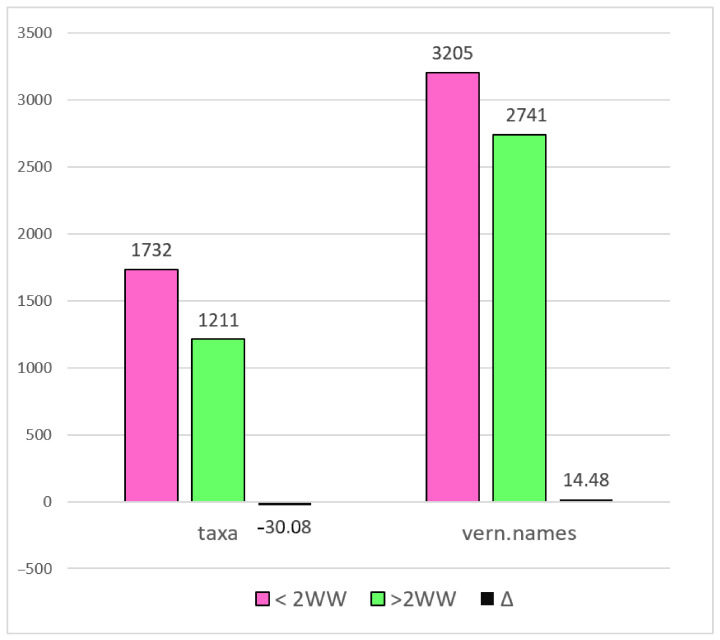
Differences in number of species belonging to VF in the two historical periods: before (<2 WW) and after (>2 WW) the 2° World War in Liguria. The variation (Δ) between the two periods is reported.

The differences in the number of species belonging to VF in the two historical periods (before and after the 2° World War) point out that there was a decrease in both the cited species (−30%) and the number of names used (−14.48%) throughout Liguria (Figure 3).

**Figure 4:**
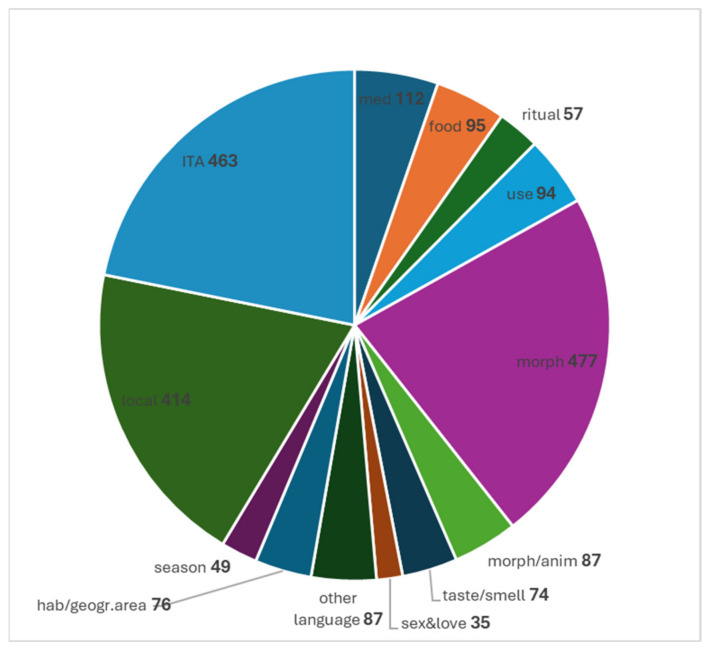
Phytonyms of vernacular names in Liguria. The thirteen categories are: ITA = of Italian derivation; local = undefined etymology and probable of regional use; season = linked to season/time flowering period; hab/geogr.area = describing the living habitat or geographical provenience; other language = deriving from foreign languages; sex and love = referred to use/morphs resembling man/woman relationship; taste/smell = linked to the senses experience with the plant; morph/anim = referring to a particular characteristic of an animal; morph = related to a generic plant morphology; use = related to human use; ritual = related to ritual/religious use; food = related to human food; med = related to medicinal use.

**Figure 5:**
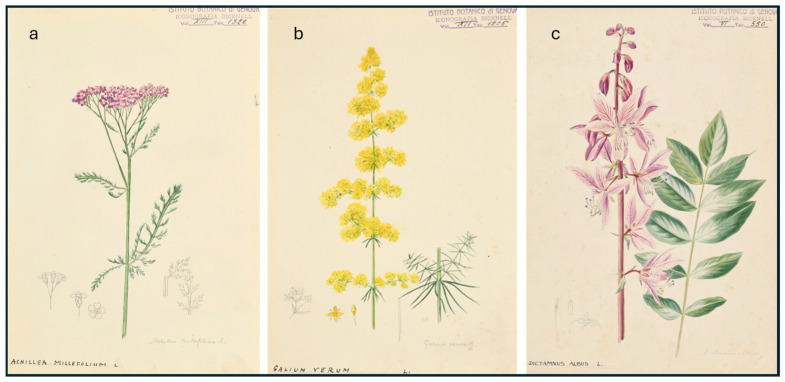
Examples of species whose vernacular name belongs to a specific category of phytonyms: (**a**) *Achillea millefolium* L. (*stagnasangue* = stopping blood—used to stop bleeding) for medicinal use; (**b**) *Galium verum* L. (*caìu* = used as rennet) for practical use; (**c**) *Dictamnus albus* L.—dittamo in Italian) of Italian derivation. Watercolors painted by Clarence Bicknell (1842–1918) and conserved at Genova University.

Based on the selected criteria, the basic plant names of VF were grouped into thirteen phytonym categories (Figure 4), as follows:

1. med (medicinal use): n = 112 [i.e., *brónchiolin-a* (bronchiolin)—*Pallenis spinosa* (L.) Cass.—used as an expectorant; *erba dei cantanti* (singers’ herb)—*Sisymbrium officinale* (L.) Scop.—used to strengthen the voice; *stagnasangue* (stopping blood)—*Achillea millefolium* L.—used to stop bleeding (Figure 5a)];
2. food: n = 95 [i.e., *buraxa sarvèga* (wild borage)—*Symphytum officinale* L.—used as a substitute for borage; *pansutti* (pansotti)—*Ficaria verna* Huds.—used to stuff fresh local pasta];
3. ritual (and religious): n = 57 [i.e., *òcci da madonna* (Madonna eyes)—*Veronica persica* Poir.—flowers interpreted as Mary’s eyes; *sàngue d’u Signú* (blood of Lord)—*Dactylorhiza sambucina* (L.) Soó—flower color interpreted as the blood of Christ; *erba stria* (witch’s weed)—*Ruta chalepensis* L.—plant used in magical rituals];
4. use (practical uses): n = 94 [i.e., *fiaschéta* (flask)—*Lagenaria siceraria* (Molina) Standl.—used as a small container for liquids; *sgarzu* (carder)—*Dipsacus fullonum* L.—to card wool; *caìu* (rennet)—*Galium verum* L.—used to curdle milk (Figure 5b)];
5. morph (morphological traits): n = 213 [i.e., *bochi* (spines)—*Rubus ulmifolius* Schott—referring to the thorns of the plant; *cinquenervi* (five nerves)—*Plantago lanceolata* L.—referring to the five leaf veins];
6. morph/anim: essentially a subcategory of the previous one, that refers to a particular characteristic of an animal, n = 87 [i.e., *u’egge d’ase* (donkey ear)—*Pulicaria dysenterica* (L.) Bernh.—for leaf shape; *bocca de gallina* (mouth of hen); *lengua de bò* (ox tongue)—*Anchusa azurea* Mill.—rough surface of the leaf];
7. taste/smell: n = 74 [i.e., *erba spùssia* (smelly grass)—*Helleborus foetidus* L.—due to the bad smell of the plant; *erba peverîna* (pepper herb)—*Satureja montana* L.—the chewed leaf tastes like pepper];
8. season: n = 49 [i.e., *sciû de mazu* (May flower)—*Centaurea cyanus* L.—flowering period of the plant; *ventidùe* (twenty-two)—*Mirabilis jalapa* L.—usually flowering in the evening];
9. hab/geogr. area (living habitat or geographical provenience): n = 76 [i.e., *erba da funtana* (fountain grass)—*Adiantum capillus-veneris* L.—typical habitat in which the plant lives; *rœsa da chin-a* (China rose)—*Alcea rosea* L.—area of origin of the species]
10. sex and love: n = 35 [i.e., *amu cornüu* (cuckold love)—*Delphinium consolida* L.—for the spur of the corolla; *galanti* (girl/boyfriends)—*Avena sativa* L.—children’s game launching spikelets to someone to figure out the number of girl/boyfriends he/she has];
11. other language (foreign linguistic contamination): n = 87 (i.e., *canderée* (little gray)—*Verbascum thapsus* L.—from the French word *cenderée*; *bricòcculu* (apricot)—*Prunus armeniaca* L.—from the Arabic *al-barcuc*; *ua dë cùch* (mountain grape)—*Ribes rubrum* L.—from the occitane word *cùch*];
12. local (regional names): n = 414 [i.e., *arbœgin-e*—*Lathyrus aphaca* L.; *cornabüggia*—*Origanum vulgare* L.];
13. ITA (Italian derivation): n = 463 [i.e., *dittamu* (burning bush)—*Dictamnus albus* L.—Italian dittamo (Figure 5c); *póro* (leek)—*Allium ampeloprasum* L.—Italian porro].

## 3. Discussion

Our study sought to assess how folk botanical knowledge from Liguria (northern Italy) has been preserved or modified over a 120-year period. In this time—from the late nineteenth century to the present day—there have been epochal historical events (two world wars), and many socioeconomic and land use changes have taken place. Since the mid-20th century, the province of Genova has seen considerable growth in the steel and metalworking industries (e.g., Ansaldo s.p.a., Piaggio s.p.a.), followed by the shipbuilding and port sectors. Therefore, the traditional villa landscape has been radically transformed, rapidly taking on the typical characteristics of industrial cities, with the consequent disappearance of peri-urban vegetable gardens [24]. Similarly, the Savona area, mainly in the Bormida Valley, has been characterized by intense industrial activity, with steel and petrochemical complexes. In the province of La Spezia, the economy has focused on the military sector (Military Arsenal) and shipbuilding. By contrast, the province of Imperia has always had a less pronounced industrial footprint, maintaining a strong bond with the territory due to its vocation for agriculture and floriculture [25]. Furthermore, during the same period, the entire region became an increasingly sought-after destination for mass-tourism, thanks to its scenic beauty and mild climate [26]. Therefore, our investigation covers a very long period, during which deep transformations have influenced the relationship between humans and plants.

Moreover, different methodologies were used to collect data by different authors, not always consistent within the broad time span considered. All these factors make it difficult to obtain a clear comparison between ethnobotanical data collected before and after the First and Second World Wars. Nevertheless, although the results obtained are purely indicative, they seem to suggest that since the mid-20th century socioeconomic, land use changes as well as the growing tourism in Liguria have contributed to reducing the population’s reliance on wild plants, thereby diminishing knowledge regarding their vernacular names. This is in agreement with the recent study of Sulaiman et al. [27], showing that similar transformations in different coastal Mediterranean areas have contributed to accelerating the erosion of local ethnobotanical knowledge (LEK), diminishing community reliance on wild flora.

However, despite the problems of the different methodologies and diverse objectives pursued over time in the folk botanical investigations [13], this legacy is too valuable to be overlooked. Therefore, we critically analyzed all documents produced by botanists recording the vernacular names of plants in Liguria (northern Italy) over a 123-year timeframe to preserve an important biocultural heritage. The picture that emerges is mainly a comprehensive assessment over the entire period of Ligurian vernacular names for 950 taxa, for which the category of use was often reported. These data had never been consolidated into a summary overview spanning more than a century.

Regarding the EF, although the taxa have been cited for multiple uses, medicinal and food uses predominate, followed by domestic purposes, and uses in the agropastoral world, including both veterinary and agricultural-related craftsmanship. These findings are also confirmed by data from the Tuscan Ethnobotanical Center (CET) for Italy in the period between 1970 and 2006: out of 1492 species used in various sectors, medicinal and food uses are the most numerous [28].

Among families most frequently reported there are Asteraceae, Fabaceae, Lamiaceae and Rosaceae. The predominance of these botanical families is common to numerous ethnobotanical surveys carried out both in Italy [29,30,31] and in other the Mediterranean countries, such as in Catalonia [32,33], areas characterized by similar floristic composition and environments, ranging from the coast of the Mediterranean basin to imposing mountains, such as the Alps or the Pyrenees [34,35]. The widespread use in traditional medicine in the Mediterranean area of plants belonging to the Lamiaceae, Asteraceae and Rosaceae families has also been confirmed by the study of Gonzalez-Tejero et al. [36] and could be representative of floristic homogeneity. The same authors suggested that it is also conceivable that ethnobotanical knowledge relating to species easily recognizable by their showy flowers, intense aromas, or characteristic flavors has contributed to the preservation of a shared cultural heritage.

Regarding the most well-known and used plants resulting from our study, there were trees such as *Castanea sativa* Mill. and *Olea europaea* L., small trees like *Juniperus communis* L. and *Sambucus nigra* L., but also the evergreen aromatic shrub *Salvia rosmarinus* Schleid and the tall, bamboo-like perennial grass *Arundo donax* L.

*C. sativa* Mill. and *O. europaea* L. have represented staples of the local agricultural tradition throughout Liguria for centuries and have been exploited as sources of wood but also for the nutritional value of their fruits and derivatives, which have always been used in the preparation of numerous typical dishes [37]. As for the olive tree, both the leaves and the oil have also been valued in the past and still today for their healthful properties, and are used for medicinal purposes, from the east to the west of Liguria [38,39,40]. In Liguria, chestnut wood was also widely used in various fishing-related crafts, to make various tools, fish traps, to dye fishing nets, and for shipbuilding [19,37].

Juniper wood has been widely used for craft purposes to make various tools. In the past, at Christmas, its branches were burned to purify and perfume homes, and there was a widespread tradition of using it as an indoor Christmas tree [41]. Furthermore, its fruits are still used today to flavor rabbit and wild boar meat, as well as grappa [39], but it is above all the medicinal use of juniper berries, with antiseptic properties [42], that has made this species highly appreciated throughout Italy: in particular, the balsamic and bechic properties of their decoction were reported in the Ligurian Apennines [43].

*S. nigra* L. has been widely used in the whole Region for different medicinal purposes as well as to prepare vinegar, syrup, jams or as ingredient of fritters. *S. rosmarinus* Schleid has always been a very common plant in home gardens or grown in pots on balconies, used to flavor various dishes and for medicinal purposes as an antispasmodic, digestive and diuretic [39,40]. Therefore, it is not surprising that these plants have maintained a predominant role over time throughout Liguria.

On the other hand, the use of *Arundo donax* L. has been reported mainly in the past, when it was widely used for its fiber in crafts, to make brooms, mats, baskets, or for its stem used as a support for cultivated plants, to create fences and pergolas, and even to make reeds to support the plaster of ceilings [44]. In Val di Vara (La Spezia), the plant was also used for the construction of toy rifles for children [41]. On the other hand, the importance of this species in the traditional medicine has continued until more recent times: from the west to the east of Liguria, the decoction of the stem parenchyma macerated with sugar, as well as the decoction of the rhizome, have been used to treat various respiratory ailments; the rhizome was also quoted for its depurative and diuretic properties, or as an antiarthritic remedy [41,45,46,47,48]. Nowadays, although the ethnobotanical uses of *A. donax* L. are generally less common than in the past, its vernacular name (*cana*) is still well known among the Ligurian population, probably also due to the widespread diffusion of the plant in the area.

Overall, our data show that traditional knowledge of local useful plants, measured by EI, has a regional value of 20.03, which is above the range of values (from 14.24 to 17.57) reported for Italy [4,30]. On the other hand, our data agree with the EI of 23.75 reported for Montecorvino (Campania, Italy) [49]. The high EI found in our study indicates that a good level of folk knowledge of plants has been maintained in Liguria, comparable to that of other Italian areas, such as Campania, characterized by similar environmental conditions. However, even higher values have been reported in other Mediterranean countries: EI of 29.01 for Pallars, Catalonia [50], 25.3 for the Cabo de Gata-Níjar, Almería (Spain) [51], and 27.7 for Tunisia [52,53].

If we consider instead the different areas of Liguria, a variability has been found in the traditional use of the local flora, more marked in the central area (C) where, during the period under study, more intense industrial and port activities developed. This fact has probably negatively affected the human-nature relationship, leading to a depletion of the use of local flora, even though in the same zone C the NF showed higher values than in W and E. As regards EI referred only to plants with medicinal use (EImed), a similar trend was observed, but a very higher value concerning the use of medicinal plants in the western zone respect to the other two parts of the territory and to the whole region was detected. In western Liguria, industrial development and port activity have always been less significant, while the horticultural sector gradually strengthened. This tradition dates to 1895, when Riviera’s first flower market opened in Ospedaletti (IM). However, it was only at the beginning of the century that the first greenhouses were built, and in the following years, the businesses transformed from artisanal to industrial, with floriculture reaching its peak around 1970 [25]. This has probably allowed for the maintenance of a closer link with the knowledge relating to the uses of plants, including those employed for medicinal purposes.

Regarding the richness of popular knowledge on wild species, the Ethnophytonomic Index (EPI) shows a very high value, always around 0.30 both for the whole region and its subdivisions. This value is in fact much higher than those of recorded in some bio-cultural refugia of the Italian Alps, such as 0.10 in the Stelvio National Park in Lombardy [54], 0.10 in South Tyrol [55] and about 0.13 in the Gran Paradiso National Park in Aosta Valley [56]. Our value is, instead, more similar to those reported for some region of the Iberian Peninsula: EPI of 0.19 in Les Guilleries (Catalogna) [33] and 0.18 in the province of Castelló [57,58].

The fact that 32.27% of wild taxa were found to be well known enough to have a vernacular name is indicative of a good conservation of plant knowledge, but it is also a consequence of the high biodiversity of Ligurian flora [59]. These data seem particularly significant if we consider that in the Bulgarian glossary by Achtarov [60], one of the most important sources for this matter, the number of plants having folk names is about 23%.

The VF identified that there was not a clear connection between the richness of names and ethnobotanical uses. Indeed, the taxa associated with more than 30 names are very common species, mostly wild greens or cultivated plants. The highest ratio of vernacular names to uses was detected for *Reichardia picroides* (L.) Roth, one of the most important components of the group of edible wild plants, collected to obtain the typical mixture of “prebuggiun” (*erbette*), used throughout Liguria for the preparation of traditional recipes [39,40,41].

The abundance of vernacular names in the western part of the region is linked to a stronger human/nature relationship and to its neighbor position with other cultures (French, Occitane and Brigasque). However, we observed for the whole of Liguria differences in number of species belonging to VF in the two historical periods, before and after the 2° World War, evidencing a decrease both in cited taxa (−30%) and in number of vernacular names used (−14.48%). In such a long and complex historical period, the Ligurian landscape underwent radical transformations, with the abandonment of inland areas and the consequent agricultural decline, and there was a shift towards massive urbanization and increased tourism. All this has led not only to a loss of landscape identity but also of much knowledge about plants, including their vernacular names and traditional uses. It should be remembered, however, that the research was conducted using different methods in the two historical periods considered and that before the war it was mainly a collection of vernacular names [61], while after the war, in addition to the collection of vernacular names of plants [23,62], greater attention was paid to their various traditional uses (for complete references see Table A2 in Appendix A).

It should also be remembered that the vernacular names of plants are generally among the last elements to be lost, even if a process of cultural erosion is markedly taking place [33]. For this reason, it is frequent that some informants remember the vernacular name of many plants but have forgotten the uses that previous generations made of them. Similarly, in our survey, we have collected the vernacular names of 950 taxa, but only 628 were the plants whose use was reported in the examined literature. Starting from the 9724 vernacular names recorded from literature, we realized that many were the cases of cross-citations among scientific papers mainly from the two historical studies on Ligurian vernacular names constituting a milestone for all recent studies (Table A2 in Appendix A). The final reduction to 6467—achieved by eliminating duplicates and names with multiple cross-references—and the exclusion of local dialectal variants or alterations resulted in a definitive count of 2120 basic regional phytonyms.

Finally, our study gives us a more detailed phytonyms analysis than previously performed because the Liguria region was, along history, a crossroad of cultures and traditions (Ligurians, naval trade with the entire Mediterranean basin, political variations). For example, the proximity to France to the west is reflected in some names, such as those commonly used along the Franco-Italian border, such as *saussiè*—*Morus alba* L.; *serfuèy*—*Anthriscus cerefolium* (L.) Hoffm.; *spinoard*—*Spinacia oleracea* L., derived from French. On the other hand, the proximity to the Tuscany region to the east is shown by other vernacular names also used in that region (*razole*—*Smilax aspera* L.; *rógna*—*Euphorbia* L.). From the Brigasque (brigašc), an Alpine Ligurian variety spoken in the Brigasca region, straddling Italy (IM/CN) and France (Maritime Alps), derives other vernacular names such as *tèŕvlacchi*: *Centranthus ruber* (L.) DC.; *xlapàss*: *Arctium lappa* L.; *ŕuš d’laŕs*: *Larix decidua* Mill.; and *ŕ šgurin*: *Salix caprea* L. used at Verdeggia.

## 4. Materials and Methods

### 4.1. Study Area and Its Natural Flora

The Liguria region is 5400 square kilometers wide and located in the northwestern part of Italy. According to the latest floristic updates, the whole Ligurian flora (Natural flora—NF) lists 3241 taxa [63,64,65,66]. All data were aligned and taxonomically complied according to Bartolucci et al. [63] and the following updates as reported at Index Plantarum Florae Italicae [67].

The NF of the region was elaborated according to the following criteria: (a) those taxa belonging to four categories (NC = no longer recorded, D = doubtfully occurring, EX = extinct, NP = recorded by mistake) [63] for which no distribution updates are available at Wikiplantbase Liguria web site were erased; (b) invasive exotic species of recent introduction were erased; (c) cultivated plants were selected and partially added in relation to their traditional uses.

### 4.2. Ethnobotanical Literature Search

A comprehensive literature search was conducted on the web sources Google, Scopus and ScienceDirect. The survey covered all available literature from database inception to March 2024. The search string was constructed using keywords and Boolean operators (AND, OR) adapted to the specific syntax of each database. The keywords used included: “Liguria”, “Ethnobotany”, “Plant names”, “Vernacular”, “Dialect”.

In addition, by using the same criteria, we conducted a manual search in the historical book collections currently preserved within the departmental office shelves of DISTAV at the University of Genova. This institutional collection comprises the legacy volumes previously held by the Library of the former Institute of Botany (Istituto di Botanica) of the same University. Relevant printed monographs, historical textbooks, and specialized botanical volumes were manually reviewed, and their reference lists were cross-checked for additional eligible sources. Eligible historical publications discovered through this process were subsequently retrieved in full text via the Library System of the University of Genova (Sistema Bibliotecario di Ateneo) and the main municipal central library of Genova, Berio Library (Biblioteca Civica Berio).

The search globally yielded a total of 30 bibliographic sources (Table A2 in Appendix A), the oldest of which dates back to the late Nineteenth century [22] while the more recent to 2020 [23]. These ethnobotanical investigations are extremely heterogeneous because they are compiled with different methods: in the past, data were collected using a network of a few local informants, while more recently a scientifically accurate method has been used, carrying out semi-structured interviews with a specific number of informants. The final aim of the research conducted in the two periods also appears to be different: before the war it was mainly a collection of vernacular names [3,22]; after the war the investigations were more thorough also with regard to the use of plants for various applications.

All data were nomenclatural aligned and taxonomically complied according to Bartolucci et al. [63] and following updates (https://www.actaplantarum.org/flora/flora.php, accessed on 11 December 2025). The data collected were used to compile different lists and analyses:

### 4.3. Ethnobotanical Flora

Ethnobotanical flora (EF) includes genera and species with a defined popular use. The plant uses were subdivided into 11 categories: food (F), medicinal (M), veterinary (V), handcraft (HC), domestic (D), agro-pastoral (AP), fishing (FS), religious (R), magical-ritual (MR), ludic (L) and poison (P). The species of the EF used for medicinal purposes were analyzed separately (EFmed).

### 4.4. Vernacular Flora and Phytonomys

Vernacular flora (VF) includes genera and species with popular name (i.e., dialectal/vernacular): data were selected eliminating repeated names in the same study; purging multiple cross-referred names quoted in different studies; detecting basic names without any local alteration and variation (e.g., *Reichardia picroides* (L.) Roth: *gattalægua*, *gatalêgua*, *lattalægua*) and names exclusively used only in a part of the region (e.g., *italiòa*, *scapiròi*).

For these reasons, we further carried out an elimination process based on different criteria, listed and explicated below with illustrative examples:

- elimination of the linguistic variations that occur in each locality of the territory, such as for *Reichardia picroides* (L.) Roth: *gattalægua*, *gatalêgua*, *lattalægua*, *ratalêgua*, *rattalægua*, *rattalégua*, *atalêgua*, *aitalêgue*, *talêgue*, *talégoa*, *lacialêgue*, *lêgue*);
- the same name used for similar plants [i.e., *scannabeccu* used for: *Centaurea calcitrapa* L. (Genova), *Cytisus scoparius* (L.) Link (Genova), *Cytisus spinosus* (L.) Lam. (Val Bisagno), *Genista germanica* L. (Genova)] or used for similar plants or of the same genus/family [*batticristi*: *Carlina acaulis* L., *Galactites tomentosus* Moench, *Asparagus acutifolius* L., *Asparagus officinalis* L., *Silybum marianum* (L.) Gaertn.; *puràssa*: *Ornithogalum umbellatum* L., *Colchicum autumnale* L., *Charybdis maritima* (L.) Steinh, *Pancratium maritimum* L., *Scilla italica* L., *Asphodelus albus* Mill., *Narcissus tazetta* L.; *viola*: *Erysimum cheiri* (L.) Crantz, *Matthiola incana* (L.) W.T.Aiton, *Viola* L. sp.pl.];
- the same name given to plants with the same use [*stagnasangue* (with haemostatic properties): *Achillea millefolium* L. and *Pilosella officinarum* F.W.Schultz & Sch.Bip.; *erba da freve* (febrifuge): *Lycopus europaeus* L. and *Centaurium erythraea* Rafn.; *erba pe’ sciatiche* (which cures sciatica): *Ranunculus bulbosus* L., *Ficaria verna* Huds., *Ranunculus aconitifolius* L. and *Ranunculus acris* L.];
- the same name used for plants with the same harvesting period, often concomitant with religious celebrations: [*erba di San Pietro*: *Hypericum perforatum* L., *Tanacetum balsamita* L. and *Carlina acaulis* L.; *erba dell’Ascensione*: *Helichrysum italicum* (Roth) G.Don, *Sedum dasyphyllum* L. and *Umbilicus rupestris* (Salisb.) Dandy; *erba da Madonna*: *Stachys recta* L., *Agrimonia eupatoria* L., *Dittrichia viscosa* (L.) Greuter, *Ajuga reptans* L., *Plantago major* L. and *Salvia verbenaca* L.].

The last value was considered as the number of regional basic phytonyms. The valuation of these phytonyms, as similarly performed by Chiocchio et al. [5], let to subdivide them into thirteen groups as follows: ITA = names of Italian derivation; local = undefined etymology and probable of regional use; season = name linked to season/time flowering period; hab/geogr.area = name describing the living habitat or geographical provenience; other language = name deriving from foreign languages/dialect; sex and love = name referred to use/morphs resembling man/woman relationship; taste/smell = name linked to the senses experience with the plant; morph/anim = name of plant refers to a particular characteristic of an animal; morph = name related to a generic plant morphology; use = name related to human use; ritual = name related to ritual/religious use; food = name of human food; med = name related to medicinal use.

All data belonging to NF, EF and VF were subdued to a geographical distribution analysis by considering the Ligurian territory sub-divisible in three parts: western (W—Imperia administrative province), central (C—Savona and Genova administrative provinces) and eastern (E—La Spezia administrative province). Further, VF was examined from a historical point of view: the species were counted as being reported in scientific papers belonging to periods before and after the Second World War.

### 4.5. Floras Analysis and Indexes

To better evaluate the three databases, the following indexes were calculated:

- Reported Use Value (RU) indicating the total number of specific uses mentioned for each plant, calculated on the taxa of EF [68].
- Ethnobotanicity Index (EI) [69] indicating the traditional use of local species, expressed as a percentage between the number of spontaneous plants used (EF) and the total number of species of the flora of the territory considered (NF).
- Ethnobotanicity Index of medicinal plants (EImed) [69] indicating the traditional local use of medicinal plants, expressed as a percentage between the number of medicinal plants used (EFmed) and the total number of species of the flora of the territory considered (NF).
- Ethnophytonomic Index (EPI) [33] expressed as a rate between the number of plants species with dialectal names (DF) and the total number of species of the flora of the territory considered (NF), indicating the traditional knowledge of local species.

The indexes were calculated both for the region as a whole and the geographical subdivision reported before (W, C and E Liguria), with the intention of detecting any variation throughout the region.

## 5. Conclusions

Our study has consolidated the data on the vernacular names of plants in Liguria into an exhaustive summary spanning more than a century. The high Ethnophytonomic Index (0.30) underscores the region’s significant botanical knowledge, particularly in the western area, where a strong tradition of floriculture and agriculture has helped to maintain robust ties to the land. However, considering Liguria as a whole, since the mid-20th century (after World War II), a 30% decline in cited species and a 14.48% reduction in recorded vernacular names have been observed.

Although historical data on folk botanical knowledge were collected using inconsistent methodologies over time, they suggest that socioeconomics, land use changes and the rapid growth of tourism—occurring over such a long and complex period—may have contributed to reducing the population’s reliance on wild plants, thereby also diminishing knowledge of their vernacular names.

By systematizing over 120 years of data, this research provides a critical baseline for the preservation of Liguria’s linguistic and ethnological diversity, serving as a tool to safeguard this fragile cultural heritage for future intergenerational transmission.

## Figures and Tables

**Table 1:** More used families of the Ethnobotanical flora (EF).

	Liguria	W	C	E
Taxa with uses	628	487	358	298
n° Fam	122	112	102	96
n° Fam > 10 uses	27	27	27	27
Amaryllidaceae	16	10	6	8
Apiaceae	31	26	9	9
Asparagaceae	17	12	9	9
Asteraceae	119	95	49	59
Boraginaceae	14	10	4	6
Brassicaceae	30	20	13	12
Cannabaceae	16	11	8	10
Caprifoliaceae	11	9	2	7
Caryophyllaceae	17	13	8	6
Chenopodiaceae	10	8	2	4
Crassulaceae	10	8	4	6
Ericaceae	13	12	6	5
Euphorbiaceae	10	7	7	7
Fabaceae	60	42	26	25
Geraniaceae	11	1	6	3
Iridaceae	10	7	4	5
Lamiaceae	56	47	25	24
Malvaceae	13	8	6	7
Oleaceae	10	7	6	8
Orchidaceae	13	7	1	1
Plantaginaceae	19	16	6	7
Poaceae	43	24	24	22
Polygalaceae	17	13	6	7
Ranunculaceae	27	24	10	11
Rosaceae	53	47	27	24
Salicaceae	10	10	5	6
Solanaceae	14	13	7	6

The families having applications >10 are reported. In bold those with applications >40. All data are reported for the region (Liguria) and the three geographical subdivisions (W, C and E Liguria).

**Table 2:** Values of NF (Natural Flora), EF (Ethnobotany Flora), VF (Vernacular Flora) and related indexes.

	NF	EF	VF	EFmed	EI	EImed	EPI
Liguria	3135	628	950	529	20.03	16.87	0.30
W	1772	488	577	404	27.54	22.80	0.33
C	2172	362	705	271	16.67	12.48	0.32
E	1514	298	442	203	19.68	13.41	0.29

EFmed = number of EF plants used for medicinal purposes. EI (Ethnobotanicity Index = EF/NF%). EImed (Ethno-med-botanicity Index = EFmed/NF%). EPI (Ethnophytonomic Index = VF/NF). All data are reported for the region Liguria and the three geographical subdivisions (W, C and E).

**Table 3:** List of the 25 taxa with more than 30 vernacular names.

Taxon	n Paper	n Citations	n Vernacular Names	n Uses	Rate N/U
*Taraxacum* F.H.Wigg. sect. *Taraxacum*	24	114	75	33	2.27
*Sonchus oleraceus* L.	16	80	62	22	2.82
*Reichardia picroides* (L.) Roth	13	71	61	16	3.81
*Laurus nobilis* L.	22	78	58	29	2.00
*Plantago major* L.	21	60	56	20	2.80
*Clematis vitalba* L.	15	74	55	35	1.57
*Plantago lanceolata* L.	19	57	47	25	1.88
*Rubus ulmifolius* Schott	19	58	45	25	1.80
*Helichrysum italicum* (Roth) G.Don	18	70	44	19	2.32
*Thymus vulgaris* L.	20	61	43	28	1.54
*Borago officinalis* L. *	26	65	40	43	0.93
*Foeniculum vulgare* Mill. *	22	56	40	42	0.95
*Parietaria officinalis* L.	24	66	40	27	1.48
*Centranthus ruber* (L.) DC.	17	48	39	18	2.17
*Allium cepa* L. *	22	59	37	26	1.42
*Fragaria vesca* L. *	13	48	37	19	1.95
*Smilax aspera* L.	13	41	37	13	2.85
*Juniperus communis* L.	15	47	35	34	1.03
*Papaver rhoeas* L.	22	56	35	22	1.59
*Vaccinium myrtillus* L.	11	40	34	22	1.55
*Arbutus unedo* L.	13	43	33	26	1.27
*Prunus avium* (L.) L. *	15	39	32	22	1.45
*Hypericum perforatum* L.	23	44	31	33	0.94
*Cichorium intybus* L.	18	39	30	29	1.03
*Petroselinum sativum* L. *	18	48	30	24	1.25

Taxa indicated with * are cultivated. The total taxa were 950 reported in the 30 reviewed bibliographic sources. The total verified citations were 8448, while the total of purged names is 6467. In the last column the rate of folk names/uses (N/U) was calculated.

## Data Availability

The data presented in this study are included in the article and in the literature reported. Further inquiries can be directed to the corresponding author.

## Supplementary Materials

The following supporting information can be downloaded at: https://www.mdpi.com/article/10.3390/plants15142193/s1, S1: Species belonging to EF and their use in Liguria for the eleven categories of different applications.

## Appendix A

**Table A1:** Ethnobotanical flora (EF) and subdivision in the eleven categories of different applications.

	F	M	V	HC	D	AP	HF	R	MR	L	P	n° sp.
TOT	255	529	144	72	203	117	18	44	65	66	62	628
W Lig	240	1066	66	41	94	72	7	15	19	29	42	487
C Lig	165	650	67	27	55	31	8	16	12	19	10	358
E Lig	232	572	72	36	131	55	8	28	54	42	16	298

F = food, M = medicinal, V = veterinary, HC = handcraft, D = domestic, AP = agro-pastoral, HF = fishing, R = religious, MR = magical-ritual, L = ludic, P = poison.

**Table A2:** Comparison of ethnobotanical literature of Liguria from 1897 to 2020.

Authors	Year	Method	Area	Tot. Species	Folk Use	Med. Plants	Vern. Names
Penzig [22]	1897	Vern names	Liguria	674	45	26	1946
Lagomaggiore & Mezzana [61]	1902	Vern names	Liguria	528	94	53	2465
Rovesti [70]	1922	Med plants	W Liguria	282	282	277	473
Bertagnon [71]	1955	Med plants	C Liguria	59	59	52	60
Chiovenda-Bensi [38]	1960	Med plants	E Liguria	53	52	44	110
Bandini [72]	1961	Med plants	E Liguria	69	69	66	84
Gastaldo et al. [73]	1979	Med plants	C Liguria	137	134	133	164
Martini [74]	1981	Med plants	C Liguria	66	66	66	66
Martini [43]	1982	Med plants	C Liguria	48	47	47	60
Villa [44]	1983	Vern names	W Liguria	150	40	10	190
Massajoli [75]	1984	Cultural	W Liguria	113	95	37	129
Viviani [76]	1991	Vern names	E Liguria	221	2	0	279
Maccioni et al. [45]	1995	Med plants	E Liguria	82	82	73	199
Minuto & Pronzati [77]	1997	Food plants	C Liguria	31	30	14	185
Oddo [78]	1997	Med plants	W Liguria	216	204	194	445
Maccioni et al. [46]	1999	Med plants	W Liguria	84	83	70	293
Marchini & Maccioni [79]	1998	Cultural	E Liguria	88	88	75	141
Marchini & Maccioni [80]	1999	Cultural	E Liguria	77	75	66	167
Maccioni et al. [81]	2000	Med plants	E Liguria	26	23	7	34
Maccioni [82]	2003	Med plants	E Liguria	41	41	41	57
Maccioni et al. [47]	2004	Ethnobot	W Liguria	61	61	61	115
Maccioni [48]	2008	Cultural	E Liguria	52	52	52	107
Camangi et al. [41]	2009	Ethnobot	E Liguria	196	171	127	785
Cornara et al. [39]	2009	Ethnobot	E Liguria	113	113	92	179
Chiarugi [83]	2012	Ethnobot	C Liguria	7	7	0	16
Cornara et al. [84]	2013	Ethnobot	C Liguria	162	132	102	237
Cornara et al. [40]	2014	Ethnobot	W Liguria	187	187	138	487
Magnozzi [85]	2014	Ethnobot	E Liguria	89	85	45	108
Maccioni [62]	2016	Vern names	E Liguria	63	63	45	84
Maccioni [23]	2020	Vern names	E Liguria	58	48	24	62

The studies are listed by year of publication, and the following data are reported: the research method used, the geographical area covered, number of species involved (total, with a folk use, medicinal) and the number of vernacular names reported.
