# Supplementary material for: Ethnobotanical Inventory on the Vernacular Names of Useful Plants: Data of over 120 Years for the Ligurian Region (Northern Italy)
Source: Plants (Basel). 2026 Jul 17;15(14):2193. doi: 10.3390/plants15142193 (PMC13417562; doi:10.3390/plants15142193)
Supplement: Supplementary file 1 [file plants-15-02193-s001.zip › plants-4406309-supplementary.pdf]

S1- Supplementary material. Species belonging to EF and their use in Liguria for the eleven categories of different applications.

Specie	F	M	V	HC	D	AP	HF	R	MR	L	P	RU
Abies alba Mill.		X		X	X			X				4
Acanthus mollis L.		X										1
Acer campestre L.		X		X	X	X						4
Achillea collina (Wirtg.) Heimerl.		X										1
Achillea erba-rotta All.		X										1
Achillea ligustica All.	X	X										2
Achillea millefolium L.	X	X			X							3
Aconitum napellus L. emend. Skalický		X				X					X	3
Actaea spicata L.		X									X	2
Adiantum capillus-veneris L.		X			X							2
Adonis aestivalis L.		X									X	2
Aesculus hippocastanum L.		X			X				X		X	4
Aethusa cynapium L.		X									X	2
Agave americana L.		X			X							2
Agrimonia eupatoria L.		X			X							2
Agrostis stolonifera L.		X										1
Ajuga chamaepitys (L.) Schreb.		X										1
Ajuga pyramidalis L.		X										1
Ajuga reptans L.		X										1
Alcea rosea L.		X			X	X						3
Allium ampeloprasum L.	X	X							X			3
Allium cepa L.	X	X			X	X			X	X		6
Allium sativum L.	X	X	X	X	X	X			X	X		8
Allium schoenoprasum L.	X											1
Allium triquetrum L.	X	X	X						X	X		5
Allium vineale L.		X										1
Alnus glutinosa (L.) Gaertn.		X		X	X	X			X			5
Aloysia citrodora Paláu	X	X			X							3
Althaea cannabina L.		X										1
Althaea officinalis L.		X			X	X						3
Amaranthus deflexus L.	X											1
Amelanchier ovalis Medik.	X				X							2
Ampelodesmos mauritanicus (Poir.) T.Durand & Schinz				X	X	X	X					5
Anchusa azurea Mill.		X										1
Anemone coronaria L.					X					X		2
Angelica sylvestris L.	X	X	X		X							4
Antennaria dioica (L.) Gaertn.		X	X		X							3
Anthemis arvensis L.		X	X		X							3
Anthericum liliago L.		X										1
Antirrhinum majus L.		X										1
Anthyllis vulneraria L.		X										1
Antirrhinum latifolium Mill.					X					X		2
Antirrhinum majus L.		X			X					X		3
Aphyllanthes monspeliensis L.				X								1
Apium graveolens L.	X	X			X							3

Aquilegia vulgaris L.	X			X								2
Arbutus unedo L.	X	X		X	X	X	X					7
Arctium lappa L.	X	X			X					X		4
Arctium minus (Hill) Bernh.		X	X		X					X		4
Arctostaphilus uva-ursi Spreng.		X	X									2
Arctostaphylos alpinus (L.) Spreng.		X	X									2
Arisarum vulgare O.Targ.Tozz.		X	X									2
Aristolochia clematis L.		X									X	2
Aristolochia rotunda L.		X								X		2
Armoracia rusticana G.Gaertn., B.Mey. & Scherb.		X										1
Arnica montana L.		X	X		X							3
Arrhenatherum elatius (L.) P.Beauv. ex J.Presl & C.Presl		X										1
Artemisia absinthium L.	X	X	X		X	X						5
Artemisia alba Turra	X	X			X				X			4
Artemisia genipi Weber ex Stechm.	X											1
Artemisia glacialis L.	X											1
Artemisia vulgaris L.		X	X									2
Arum italicum Mill.	X	X	X			X			X		X	6
Aruncus dioicus (Walter) Fernald	X	X										2
Arundo donax L.	X	X	X	X	X	X			X	X	X	9
Asarum europaeum L.		X			X						X	3
Asparagus acutifolius L.	X	X			X	X			X			5
Asparagus officinalis L.	X	X										2
Asphodelus albus L.					X							1
Asplenium adiantum-nigrum L.		X										1
Asplenium ceterach L.		X	X									2
Asplenium trichomanes L.		X	X									2
Atriplex hortensis L.	X				X							2
Atriplex prostrata Boucher ex DC.		X										1
Atropa belladonna L.		X							X		X	3
Avena fatua L.			X							X		2
Avena sativa L.		X	X	X		X				X		5
Ballota nigra L.		X										1
Barlia robertiana Greuter		X										1
Bellis perennis L.	X	X			X			X		X		5
Berberis vulgaris L.		X										1
Beta vulgaris L.	X	X	X			X						4
Beta vulgaris L. var. *rapa*	X	X				X				X		4
Betonica officinalis L.	X	X	X									3
Betula pendula Roth		X										1
Bistorta officinalis Delarbre		X										1
Bituminaria bituminosa (L.) C.H.Stirt.		X	X									2
Blackstonia perfoliata (L.) Huds.		X									X	2
Blitum bonus-henricus (L.) Rchb.	X	X				X						3
Borago officinalis L.	X	X	X	X	X	X						6
Brachypodium pinnatum (L.) P. Beauv.		X							X			2
Brachypodium sylvaticum (Huds.) P. Beauv.		X										1

Brassica nigra (L.) W.D.J.Koch		X										1
Brassica oleracea L.	X	X			X	X						4
Brassica rapa L.						X						1
Bryonia dioica Jacq.		X									X	2
Bunium bulbocastanum L.	X											1
Buxus sempervirens L.		X		X	X					X		4
Calendula arvensis (Vaill.) L.		X										1
Calendula officinalis L.	X	X			X				X			4
Calluna vulgaris (L.) Hull		X	X		X							3
Camellia japonica L.		X										1
Campanula medium L.		X			X							2
Campanula rapunculus L.	X	X				X						3
Camphorosma monspeliaca L.		X										1
Cannabis sativa L.				X	X	X			X			4
Capparis spinosa L.	X	X										2
Capsella bursa-pastoris (L.) Medik.	X	X	X							X		4
Carbenia benedicta B. et H.	X	X										2
Carduus litigiosus Nocca & Balb.		X										1
Carduus pycnocephalus L.		X										1
Carlina acanthifolia All.	X				X							2
Carlina acaulis L.	X	X			X							3
Carlina vulgaris L.		X			X							2
Carpobrotus acinaciformis (L.) L.Bolus		X										1
Carum carvi L.	X	X			X							3
Carum petroselinum B.H	X	X										2
Castanea sativa Mill.	X	X	X	X	X	X	X		X	X		9
Centaurea cyanus L.	X	X			X			X				4
Centaurea nervosa Willd.		X										1
Centaurea nigrescens Willd.		X										1
Centaurea scabiosa L.		X										1
Centaurea scabiosa subsp. *alpestris* (Hegetschw.) Nyman		X										1
Centaurium erythraea Rafn	X	X			X						X	4
Centranthus ruber (L.) DC.	X	X	X		X							4
Ceratonia siliqua L.		X	X									2
Cetraria islandica (L.) Ach.		X										1
Chamaemelum nobile (L.) All.		X										1
Chamaerops humilis L.					X			X				2
Charybdis maritima (L.) Speta		X									X	2
Chelidonium majus L.		X	X			X			X			4
Chenopodiastrum murale (L.) S.Fuentes, Uotila & Borsch	X											1
Chenopodium album L.	X					X						2
Chenopodium vulvaria L.		X										1
Chondrilla juncea L.	X											1
Cicer arietinum L.	X					X						2
Cichorium endivia L.	X	X	X			X						4
Cichorium intybus L.	X	X				X						3
Cineraria maritima L.		X										1

Cirsium arvense (L.) Scop.	X		X									2
Cirsium erisithales (Jacq.) Scop.	X											1
Cirsium vulgare (Savi) Ten.		X										1
Cistus albidus L.		X		X	X							3
Cistus creticus		X			X							2
Cistus monspeliensis L.		X			X							2
Citrus aurantium L. var. *amara* L.		X				X						2
Citrus limon (L.) Osbeck	X	X				X						3
Citrus x *aurantium* L.	X	X										2
Clematis flammula L.	X	X			X						X	4
Clematis vitalba L.	X	X	X	X		X				X	X	7
Clinopodium nepeta (L.) Kuntze subsp. *nepeta*	X	X										2
Colchicum autumnale L.		X				X					X	3
Conium maculatum L.		X									X	2
Convallaria majalis L.		X			X							2
Convolvulus althaeoides L.		X										1
Convolvulus arvensis L.	X	X	X									3
Convolvulus sepium L.		X										1
Coriaria myrtifolia L.		X	X								X	3
Cornus mas L.	X	X		X								3
Corylus avellana L.	X	X		X	X	X				X		6
Crataegus laevigata (Poir.) DC.		X			X	X						3
Crataegus monogyna Jacq.	X	X		X	X	X			X			6
Crepis biennis L.	X											1
Crepis leontodontoides All.	X											1
Crepis vesicaria L. subsp. *taraxacifolia* (Thuill.) Thell.	X											1
Crithmum maritimum L.	X	X	X									3
Crocus ligusticus Mariotti		X				X						2
Crocus vernus (L.) Hill					X							1
Cucurbita maxima Duchesne	X	X		X	X	X				X		6
Cucurbita pepo L.	X	X			X	X						4
Cupressus sempervirens L.	X	X			X							3
Cuscuta epithymum (L.) L.		X										1
Cyclamen hederifolium Aiton					X							1
Cyclamen purpurascens Mill.		X										1
Cydonia oblonga Mill.	X	X			X							3
Cynara cardunculus L.		X										1
Cynara cardunculus subsp. *scolymus* (L.) Hegi	X		X									2
Cynodon dactylon (L.) Pers.		X	X		X							3
Cynoglossum officinale L.		X										1
Cyperus esculentus L.	X											1
Cytisophyllum sessilifolium (L.) O. Lang		X										1
Cytisus hirsutus L.			X		X							2
Cytisus scoparius (L.) Link		X		X	X	X						4
Dactylorhiza maculata (L.) Soó subsp. *fuchsii* (Druce) Hyl.		X										1
Dactylorhiza sambucina (L.) Soó		X										1

Daphne gnidium L.		X					X				X	3
Daphne laureola L.		X					X		X			3
Daphne mezereum L.		X									X	2
Datura stramonium L.		X				X			X		X	4
Daucus carota L. subsp. *carota*	X	X	X		X					X		5
Delphinium consolida L.		X										1
Dianthus carthusianorum L.		X			X							2
Dianthus seguieri Vill. subsp. *seguieri*		X										1
Dictamnus albus L.	X	X										2
Digitalis ambigua Murr.		X										1
Digitalis lutea L.		X				X				X	X	4
Digitaria sanguinalis (L.) Scop.			X									1
Dioscorea communis (L.) Caddick & Wilkin	X	X									X	3
Diplotaxis muralis (L.) DC.	X											1
Diplotaxis tenuifolia (L.) DC.	X											1
Dipsacus fullonum L.	X	X			X							3
Dittrichia graveolens (L.) Greuter		X			X							2
Dittrichia viscosa (L.) Greuter		X		X								2
Dracunculus vulgaris Schott		X										1
Drosera rotundifolia L.		X										1
Dryas octopetala L.		X										1
Dryopteris filix-mas (L.) Schott		X	X		X	X						4
Ecballium elaterium (L.) A.Rich.		X										1
Echium vulgare L.	X	X										2
Elymus repens (L.) Gould		X	X									2
Emerus major Mill.		X				X						2
Equisetum arvense L.		X	X		X	X						4
Equisetum telmateia Ehrh.		X	X		X	X				X		5
Erica arborea L.		X		X	X	X		X	X			6
Erica carnea L.		X										1
Erica cinerea L.		X										1
Erica scoparia L.		X		X	X				X			4
Erigeron canadensis L.		X										1
Erodium botrys (Cav.) Bertol.										X		1
Erybotria japonica (Thunb.) Lindl.	X	X	X									3
Eryngium campestre L.		X										1
Eucalyptus camaldulensis Dehnh.		X			X	X						3
Eupatorium cannabinum L.		X										1
Euphorbia characias L.							X					1
Euphorbia cyparissias L.		X					X				X	3
Euphorbia dendroides L.		X				X	X					3
Euphorbia helioscopia L.		X	X								X	3
Euphorbia lathyris L.		X			X	X	X				X	5
Euphorbia spinosa L.		X	X									2
Euphrasia officinalis L.		X										1
Fagus sylvatica L.	X	X	X	X	X							5
Ficaria verna Huds.	X	X	X								X	4
Ficus carica L.	X	X	X	X		X						5

Foeniculum vulgare Mill.	X	X	X	X	X	X			X	X		8
Foeniculum vulgare Mill. subsp. *piperitum* (Ucria) Bég.	X	X										2
Fragaria vesca L.	X	X			X			X		X		5
Frangula alnus Mill.		X										1
Fraxinus excelsior L.		X	X	X		X						4
Fraxinus ornus L.		X	X	X	X	X				X		6
Fumaria capreolata L.	X	X				X						3
Fumaria officinalis L.		X										1
Galactites tomentosus Moench	X											1
Galatella sedifolia (L.) Greuter		X										1
Galium aparine L.		X				X						2
Galium verum L.		X										1
Genista cinerea (Vill.) DC.			X	X								2
Genista pilosa L.						X						1
Gentiana acaulis L.	X	X										2
Gentiana asclepiadea L.		X										1
Gentiana ligustica R.Vilm. & Chop.	X	X	X									3
Gentiana lutea L.	X	X		X								3
Gentianella campestris (L.) Börner	X	X		X								3
Geranium robertianum L.		X										1
Geranium rotundifolium L.		X	X									2
Geranium sanguineum L.			X									1
Geum urbanum L.	X	X										2
Globularia alypum L.		X										1
Globularia bisnagarica L.		X										1
Hedera helix L.		X	X		X			X	X			5
Helianthus tuberosus L.		X										1
Helichrysum italicum (Roth) G.Don	X	X	X		X		X		X			6
Helichrysum stoechas (L.) Moench	X				X	X						3
Helleborus foetidus L.		X	X		X		X		X			5
Helleborus niger L.		X	X				X				X	4
Helleborus viridis L.		X	X		X		X		X	X	X	7
Helminthotheca echiodes (L.) Holub	X											1
Helosciadium nodiflorum (L.) W.D.J.Koch	X	X										2
Herniaria glabra L.		X										1
Hieracium tomentosum L.		X										1
Hordelymus europaeus (L.) Harz	X											1
Hordeum vulgare L.	X	X	X		X	X				X		6
Humulus lupulus L.	X	X										2
Hyacinthus orientalis L.					X							1
Hylotelephium anacampseros (L.) H. Ohba		X										1
Hylotelephium maximum (L.) Holub		X			X			X	X			4
Hyoscyamus niger L.		X									X	2
Hyoseris radiata L.	X	X	X									3
Hypericum androsaemum L.		X										1
Hypericum perforatum L.	X	X	X		X				X	X		6
Hypochaeris achyrophorus L.	X											1
Hypochaeris radicata L.	X	X			X	X						4

Hyssopus officinalis L.	X	X			X							3
Ilex aquifolium L.		X			X							2
Impatiens noli-tangere L.		X										1
Inula helenium L.	X	X										2
Iris florentina L.	X	X			X							3
Iris germanica L.	X	X			X	X		X				5
Iris lutescens Lam.					X							1
Iris pallida L.	X	X			X							3
Isatis tinctoria L.					X							1
Juglans regia L.	X	X	X	X	X	X			X	X		8
Juniperus communis L.	X	X	X	X	X	X		X	X	X		9
Juniperus oxycedrus L.	X	X	X		X							4
Laburnum alpinum (Mill.) Bercht. & J. Presl		X		X				X				3
Laburnum anagyroides Medik.		X	X	X	X	X		X			X	7
Lactuca perennis L.		X										1
Lactuca sativa L.		X										1
Lactuca sativa L. subsp. *serriola* (L.) Galasso, Banfi, Bartolucci & Ardenghi	X		X									2
Lactuca virosa L.		X										1
Lagenaria siceraria (Molina) Standl.					X							1
Lamium album L.		X										1
Lapsana communis L	X											1
Larix decidua Mill.		X		X	X							3
Lathyrus latifolius L.					X	X		X		X		4
Lathyrus sylvestris L.	X		X									2
Laurus nobilis L.	X	X			X			X	X			5
Lavandula angustifolia Mill.	X	X		X	X	X			X			6
Lavandula latifolia Medik.	X	X			X							3
Lavandula stoechas L.	X	X			X	X						4
Lavandula officinalis Chaix	X	X			X							3
Lavatera maritima Gouan.		X										1
Leontodon hispidus L.	X											1
Leontodon tuberosus L.	X	X										2
Lepidium latifolium L.		X										1
Leucanthemum adustum (W.D.J. Koch) Gremli								X		X		2
Leucanthemum vulgare (Vaill.) Lam.	X	X						X		X		4
Levisticum officinale L.		X										1
Lichen pulmonarius L.		X										1
Ligustrum vulgare L.		X			X							2
Lilium bulbiferum L. subsp. *croceum* (Chaix) Jan		X						X				2
Lilium candidum L.		X			X			X				3
Linaria vulgaris Mill.		X			X							2
Linum suffruticosum L. subsp. *salsoloides* (Lam.) Rouy		X										1
Linum usitatissimum L. subsp. *angustifolium* (Huds.) Thell.		X	X	X		X		X				5
Linum viscosum L.		X										1
Lithospermum officinale L.		X										1

Lolium temulentum L.			X									1
Lonicera caprifolium L.		X			X							2
Lotus corniculatus L.		X										1
Lunaria annua L.					X							1
Lupinus albus L.	X	X	X			X						4
Lycopodium clavatum L.		X	X									2
Lycopus europaeus L.		X										1
Lysimachia arvensis (L.) U.Manns & Anderb.		X										1
Lythrum salicaria L.		X										1
Malus sylvestris (L.) Mill.	X	X	X	X	X	X			X			7
Malva arborea (L.) Webb & Berthel.	X	X	X							X		4
Malva neglecta Wallr.		X										1
Malva pusilla L.		X										1
Malva sylvestris L.	X	X	X		X							4
Marrubium vulgare L.		X										1
Matricaria chamomilla L.	X	X	X		X					X		5
Matthiola incana (L.) W.T.Aiton		X						X				2
Medicago sativa L.		X	X									2
Melissa officinalis L.	X	X			X	X						4
Melittis melissophyllum L		X										1
Mentha aquatica L.	X	X			X							3
Mentha longifolia (L.) L.	X	X										2
Mentha pulegium L.	X	X			X							3
Mentha spicata L.		X			X							2
Mentha suaveolens Ehrh.		X										1
Mentha x *piperita* L.	X	X			X							3
Mercurialis annua L.		X	X		X						X	4
Mespilus germanica L.	X	X							X			3
Meum athamanticum Iacq.		X										1
Moehringia muscosa L.		X										1
Momordica charantia L.		X										1
Morus alba L.	X											1
Morus nigra L.		X		X								2
Muscari comosum (L.) Mill.	X	X										2
Muscari neglectum Guss. ex Ten.					X					X		2
Myosotis ramosisssima Rochel		X										1
Myrtus communis L.	X	X		X	X				X			5
Narcissus poeticus L.		X			X							2
Narcissus pseudonarcissus L.								X				1
Narcissus tazetta L.										X		1
Nasturtium officinale R.Br.	X	X	X		X							4
Nepeta cataria L.		X										1
Nepeta foliosa Moris	X	X										2
Nerium oleander L.		X									X	2
Nicotiana tabacum L.		X										1
Nigella damascena L.	X	X			X							3
Ocimum basilicum L.	X	X			X							3
Oenanthe phellandrium Lam.		X									X	2

Olea europaea L.	X	X	X	X	X			X	X	X		8
Onobrychis viciifolia Scop.		X	X									2
Ononis spinosa L. subsp. *arvensis* (L.) Greuter & Burdet		X										1
Ophioglossum vulgatum L.		X	X									2
Ophrys fusca Link.		X										1
Ophrys holosericea W. Greuter.		X										1
Opuntia ficus-indica Mill.		X										1
Orchis mascula L.		X										1
Origanum majorana L.	X	X			X	X						4
Origanum vulgare L.	X	X			X							3
Oryza sativa L.	X	X				X			X			4
Ostrya carpinifolia Scop.		X	X	X	X							4
Oxalis acetosella L.		X								X		2
Oxalis corniculata L.	X											1
Oxalis pes-caprae L.	X	X										2
Paeònia peregrina Will		X										1
Pallenis spinosa (L.) Cass.	X	X										2
Pancratium maritimum L.											X	1
Panicum miliaceum L.		X										1
Papaver rhoeas L.	X	X	X		X			X	X	X		7
Parietaria officinalis L.	X	X	X		X							4
Paris quadrifolia L.		X									X	2
Passiflora incarnata L.		X			X							2
Pastinaca sativa L.	X	X									X	3
Pentanema squarrosum (L.) D. Gut.Larr., Santos-Vicente, Anderb., E.Rico & M.M. Mart.Ort.		X										1
Petasites hybridus (L.) G. Gaertn., B. Mey. & Scherb.		X	X			X						3
Petasites pyrenaicus (L.) G.López		X										1
Petrosedum rupestre (L.) P.V.Heath		X	X		X					X		4
Petroselinum sativum L.	X	X	X						X		X	5
Peucedanum officinale L.		X										1
Peucedanum ostruthium Koch	X	X										2
Phaseolus vulgaris L.		X				X						2
Phoenix canariensis L.								X				1
Physalis alkekengi L.		X										1
Phyteuma michelii All.	X	X										2
Phytolacca americana L.					X		X					2
Picea abies (L.) H.Karst.		X			X			X				3
Picris hieracioides L.	X	X										2
Pilosella officinarum Vaill.	X	X										2
Pimpinella anisum L.		X										1
Pimpinella saxifraga L.		X										1
Pinguicula vulgaris L.		X										1
Pinus halepensis Mill.	X	X		X	X			X				5
Pinus nigra Arnold		X		X								2
Pinus pinaster Aiton	X	X	X	X	X			X				6
Pinus pinea L.	X	X		X	X			X		X		6

Pinus sylvestris L.	X	X	X	X	X	X		X		X		8
Pistacia lentiscus L.	X	X			X					X		4
Pistacia terebinthus L.		X										1
Plantago coronopus L.	X	X										2
Plantago lanceolata L.	X	X	X							X		4
Plantago major L.	X	X	X	X						X		5
Plantago media L.	X											1
Poa annua L.	X											1
Polycarpon tetraphyllum (L.) L.		X										1
Polygala nicaeensis W.D.J.Koch		X										1
Polygala vulgaris L.	X	X										2
Polygonatum odoratum (Mill.) Druce		X			X							2
Polygonum aviculare L.		X	X				X					3
Polygonum bistorta L.		X										1
Polypodium vulgare L.	X	X								X		3
Posidonia oceanica (L.) Delile	X						X					2
Potentilla argentea L.		X										1
Potentilla erecta (L.) Raeusch.		X										1
Potentilla micrantha Ramond ex DC.									X			1
Potentilla recta L.		X										1
Potentilla reptans L.		X			X							2
Poterium sanguisorba L.	X	X										2
Primula veris L. subsp. *columnae* (Ten.) Maire & Petitm.	X	X										2
Primula vulgaris Huds.	X	X			X					X		4
Prunella vulgaris L.		X										1
Prunus avium (L.) L.	X	X		X		X				X		5
Prunus cerasus L.	X					X						2
Prunus domestica L.	X	X				X						3
Prunus dulcis (Mill.) D.A. Webb	X	X			X							3
Prunus laurocerasus L.	X	X										2
Prunus persica (L.) Batsch	X	X		X	X	X						5
Prunus spinosa L.	X	X			X	X						4
Pteridium aquilinum (L.) Kuhn		X	X		X	X		X	X			6
Pulicaria dysenterica Gaertn		X										1
Pulmonaria officinalis L.	X	X			X							3
Pulsatilla alpina (L.) Delarbre		X									X	2
Punica granatum L.		X										1
Pyrus communis L.	X	X		X		X						4
Quercus cerris L.	X	X			X	X						4
Quercus ilex L.	X	X	X	X	X	X						6
Quercus petraea (Matt.) Liebl.	X	X	X	X	X							5
Quercus pubescens Willd.	X	X			X	X						4
Quercus robur L.		X			X							2
Quercus suber L.		X			X							2
Ranunculus aconitifolius L.		X										1
Ranunculus acris L.		X	X					X			X	4
Ranunculus bulbosus L.		X	X					X			X	4
Ranunculus lanuginosus L.		X	X								X	3

Raphanus raphanistrum L. subsp. *landra* (Moretti ex DC.) Bonnier & Layens	X	X	X									3
Raphanus raphanistrum L. subsp. *sativus* (L.) Schmalh	X	X										2
Reichardia picroides (L.) Roth	X	X	X									3
Rhagadiolus stellatus (L.) Gaertn.	X											1
Rhamnus alaternus L.		X										1
Rhamnus cathartica L.		X			X							2
Rhaponticum bicknellii (Briq.) Banfi, Galasso & Soldano		X										1
Rhinanthus angustifolius C.C. Gmel.						X						1
Rhinanthus ovifugus Chabert			X						X			2
Rhododendron ferrugineum L.		X	X			X						3
Ribes petraeum Wulfen	X											1
Ribes rubrum L.	X											1
Ribes uva-crispa L.	X											1
Ricinus communis L.		X			X						X	3
Robinia pseudoacacia L.	X	X	X	X				X				5
Rosa canina L.	X	X	X		X			X	X	X		7
Rosa dumalis Bechst.	X	X									X	3
Rosa gallica L.	X	X										2
Rosa nitidula Besser		X										1
Rosa pendulina L.	X	X										2
Rosa x *damascena*	X					X						2
Rubia peregrina L.			X									1
Rubia tinctorum L.			X									1
Rubus caesius L.	X											1
Rubus hirtus Waldst. & Kit.		X										1
Rubus idaeus L.	X	X				X						3
Rubus ulmifolius Schott	X	X	X		X	X			X			6
Rumex acetosa L.		X				X				X		3
Rumex acetosella L.	X											1
Rumex conglomeratus Murray		X										1
Rumex crispus L.	X	X	X		X	X		X		X		7
Rumex obtusifolius L.		X	X		X							3
Rumex sanguineus L.		X	X									2
Rumex scutatus L.		X										1
Ruscus aculeatus L.		X			X							2
Ruta chalepensis L.	X	X							X		X	4
Ruta graveolens L.	X	X	X		X				X			5
Salix alba L.				X		X		X	X			4
Salix apennina A.K.Skvortsov				X		X						2
Salix caprea L.		X	X			X				X		4
Salix eleagnos Scop.				X								1
Salix purpurea L.				X		X						2
Salix x *fragilis* L.		X	X	X		X						4
Salvia glutinosa L.		X										1
Salvia officinalis L.	X	X			X	X					X	5
Salvia pratensis L.	X	X										2
Salvia rosmarinus Schleid.	X	X	X	X	X	X		X	X		X	9

Salvia sclarea L.	X	X			X							3
Salvia verbenaca L.	X	X										2
Sambucus ebulus L.		X			X							2
Sambucus nigra L.	X	X	X	X	X	X			X	X	X	9
Sanguisorba officinalis L.		X										1
Sanicula europaea L.		X										1
Santolina chamaecyparissus L.		X										1
Santolina ligustica Arrigoni		X			X							2
Saponaria officinalis L.		X			X						X	3
Satureja montana L.	X	X			X							3
Scabiosa columbaria L.		X										1
Scilla italica L.		X									X	2
Scolymus hispanicus L.		X										1
Scorzonera austriaca Willd.						X						1
Scrophularia auriculata L.			X									1
Scrophularia canina L.		X										1
Scrophularia nodosa L.		X										1
Secale cereale L.	X	X	X	X	X	X		X	X			8
Securigera varia (L.) Lassen		X										1
Sedum acre L.		X							X	X		3
Sedum cepae L.									X			1
Sedum dasyphyllum L.	X	X	X		X			X	X	X		7
Sempervivum montanum L. subsp. *burnatii* Wettst. ex Hayek		X										1
Sempervivum tectorum L.		X	X		X				X			4
Senecio vulgaris L.		X	X		X							3
Sherardia arvensis L.			X									1
Sideritis romana (L.) E.H.L. Krause		X										1
Silene italica (L.) Pers.	X											1
Silene latifolia Poir.	X	X			X							3
Silene vulgaris (Moench) Garcke	X									X		2
Silybum marianum (L.) Gaertn.		X										1
Sinapis arvensis L.	X											1
Sisymbrium officinale (L.) Scop.		X										1
Sixalix atropurpurea (L.) Greuter & Burdet		X										1
Smilax aspera L.	X	X			X						X	4
Solanum dulcamara L.		X								X	X	3
Solanum lycopersicum L.	X	X			X	X						4
Solanum melongena L.		X										1
Solanum nigrum L.		X				X					X	3
Solanum pseudocapsicum L.		X										1
Solanum tuberosum L.	X	X	X			X			X		X	6
Solidago virgaurea L.		X										1
Sonchus oleraceus L.	X	X	X									3
Sorbus aucuparia L.		X	X									2
Sorbus chamaemespilus (L.) Crantz	X					X						2
Sorbus domestica L.		X		X								2
Spartium junceum L.		X	X	X	X	X		X	X		X	8
Spiraea ulmaria L.		X			X							2

Stachys recta L.		X	X						X			3
Stachys romana (L.) E.H.L. Krause		X							X			2
Staphisagria macrosperma Spach		X									X	2
Stellaria media (L.) Vill.	X	X	X			X						4
Succisa pratensis Moench		X										1
Symphytum officinale L.		X										1
Symphytum tuberosum L.	X	X										2
Tanacetum balsamita L.	X	X			X							3
Tanacetum parthenium (L.) Sch.Bip.	X	X	X		X							4
Tanacetum vulgare L. subsp. *crispum* DC.	X	X	X		X							4
Tanacetum vulgare L.	X											1
Taraxacum F.H.Wigg. sect. *Taraxacum*	X	X	X							X		4
Taxus baccata L.		X										1
Teucrium chamaedrys L.		X									X	2
Teucrium marum L.		X										1
Teucrium polium L.		X										1
Teucrium scordium L.		X										1
Thymus pulegioides L.	X	X	X		X							4
Thymus serpyllum L.	X											1
Thymus vulgaris L.	X	X	X	X	X	X			X			7
Tilia americana L.		X										1
Tilia cordata L.	X	X			X							3
Tilia platyphyllos Scop.	X	X			X	X						4
Tilia x *europaea* L.		X										1
Tragopogon porrifolius L.		X										1
Trifolium alpinum L.		X										1
Trifolium pratense L.	X	X	X							X		4
Trigonella foenum-graecum L.		X										1
Trigonella officinalis (L.) Coulot & Rabaute		X										1
Triticum aestivum L.	X	X	X		X	X		X	X			7
Trollius europaeus L.			X					X				2
Tussilago farfara L.		X				X					X	3
Ulmus minor Mill.		X		X								2
Umbilicus rupestris (Salisb.) Dandy	X	X										2
Urospermum dalechampii (L.) F.W.Schmidt	X	X										2
Urtica dioica L. subsp. *dioica*	X	X	X	X	X	X			X		X	8
Vaccinium myrtillus L.	X	X				X						3
Vaccinium uliginosum L.		X										1
Vaccinium vitis-idaea L.		X										1
Valeriana officinalis L.	X	X			X							3
Valerianella locusta (L.) Laterr.	X	X										2
Veratrum album L.		X	X		X						X	4
Verbascum phlomoides L.		X										1
Verbascum thapsus L.	X	X		X	X				X	X		6
Verbena officinalis L.		X										1
Veronica allionii Vill.	X	X				X						3
Veronica anagallis-aquatica L.	X	X	X									3
Veronica beccabunga L.		X										1

Veronica chamaedrys L.		X										1
Veronica officinalis L.		X										1
Veronica persica Poir.		X										1
Viburnum tinus L.		X										1
Vicia faba L.	X		X									2
Vicia lens (L.) Coss. & Germ.	X											1
Vinca major L.		X										1
Vinca minor L.		X										1
Vincetoxicum hirundinaria Medik.		X									X	2
Viola bertolonii Pio	X	X			X							3
Viola odorata L.	X	X			X	X			X	X		6
Viola reichembachiana Jordan ex Boreau	X	X			X							3
Viola tricolor L.		X										1
Viscum album L.		X		X			X	X				4
Vitex agnus-castus L.		X										1
Vitis labrusca L.	X				X							2
Vitis vinifera L.	X	X	X	X	X	X			X	X		8
Zea mays L.	X	X	X	X	X			X	X	X		8

F = food, M = medicinal, V = veterinary, HC = handcraft, D = domestic, AP = agro-pastoral, HF = fishing, R = religious, MR = magical-ritual, L = ludic, P = poison. The RU value (is reported in the last column.
